# The Relationship Between Disease Activity and Fecal Calprotectin and Fecal Occult Blood in Inflammatory Bowel Disease: The Role of Nutritional Status

**DOI:** 10.3390/nu17213379

**Published:** 2025-10-28

**Authors:** Ali Bilgen, Hale Akpınar

**Affiliations:** 1 Department of Gastroenterology, Private Anka Hospital, Şehitkamil District, 99 Number Street, No. 162, Gaziantep 27590, Turkey; 2 Department of Gastroenterology, Faculty of Medicine, Dokuz Eylul University, İzmir 35340, Turkey

**Keywords:** ulcerative colitis, Crohn’s disease, fecal test, nutrition, disease activity, inflammation, malnutrition

## Abstract

**Background:** Inflammatory bowel disease (IBD), encompassing ulcerative colitis (UC) and Crohn’s disease (CD), is characterized by chronic intestinal inflammation with fluctuating clinical severity. Although fecal calprotectin (FC) and fecal occult blood (FOBT) are established noninvasive biomarkers of intestinal inflammation, their interplay with nutritional status and disease activity has not been fully elucidated. This study aimed to explore the relationship between FC, FOBT, and disease activity in IBD, and to assess the potential mediating role of nutritional status as measured by the prognostic nutritional index (PNI). **Methods:** This retrospective study includes 128 adult patients with confirmed IBD (50 UC and 78 CD) examined at a tertiary care center between December 2023 and August 2025. Disease activity was assessed using the Mayo score for UC and the Harvey–Bradshaw Index for CD. FC levels were quantitatively measured using an enzyme-linked immunosorbent assay (ELISA), and fecal occult blood testing was performed with an automated latex agglutination-based system. Multivariable linear regression models were conducted to identify independent predictors of disease activity. **Results:** UC patients had significantly higher FC levels (278.0 vs. 133.5 µg/g, *p* < 0.001), FOBT positivity rates (76.7% vs. 43.6%, *p* = 0.002), and lower PNI (49.2 ± 4.2 vs. 51.5 ± 4.6, *p* = 0.048) compared to CD patients. In both UC and CD, disease activity scores were positively correlated with FC, FOBT positivity, CRP, and duration of illness, and negatively correlated with PNI (*p* < 0.05). In multivariable regression, PNI lost predictive value when FC and FOBT were included; FC and FOBT remained strong independent predictors of disease activity. **Conclusions:** FC and fecal occult blood are independently associated with higher disease activity in IBD, and may mediate the observed relationship between poor nutritional status and inflammation severity. The loss of significance of PNI in adjusted models suggests that intestinal inflammation and bleeding may act as intermediaries linking malnutrition to disease activity.

## 1. Introduction

Inflammatory bowel disease (IBD) is a chronic gastrointestinal disorder characterized by periods of remission and relapse. It comprises two primary subtypes: Ulcerative colitis (UC), generally confined to the colon with inflammation typically restricted to the mucosal and submucosal layers, and Crohn’s disease (CD), which is distinguished by transmural inflammation and skip lesions that can affect any part of the gastrointestinal tract, from the mouth to the anus [1,2]. Although the precise etiology of IBD remains unclear, various genetic, microbial, familial, and environmental factors are implicated, alongside immune system dysregulation [3]. IBD severity is assessed using clinical activity indices that incorporate patient symptoms, inflammatory markers, and endoscopic findings [4].

One noninvasive approach to gauge intestinal inflammation in IBD is measuring fecal calprotectin (FC). Calprotectin is a calcium-binding protein released by activated neutrophils, and its concentration in stool rises sharply during active gut inflammation [5]. FC has become an established biomarker for distinguishing inflammatory disease from functional disorders and for monitoring IBD activity [6]. Several studies have demonstrated that FC correlates well with endoscopic disease severity in both UC and CD [6,7,8,9]. In addition to calprotectin, fecal occult blood is another stool marker that reflects intestinal disease activity. Occult blood testing (particularly immunochemical fecal occult blood tests, iFOBT) detects minute bleeding in the GI tract, which frequently occurs with active IBD due to mucosal ulceration. A positive fecal occult blood is common during IBD flares and serves as a quick, inexpensive indicator of mucosal injury [6]. Given its reduced sensitivity relative to FC and its status as a late marker of substantial intestinal hemorrhage in IBD, the fecal occult blood test is not employed in routine practice [10]. On the other hand, studies in UC have shown that serial iFOBT measurements can predict flare-ups: for about 20% of patients, a spike in fecal hemoglobin was observed weeks before clinical symptoms reappeared [11]. This suggests that rising occult blood in stool accompanies subclinical inflammation and heralds impending relapse.

Active inflammation can lead to malabsorption, reduced intake, and metabolic derangements, causing weight loss and nutrient deficiencies; conversely, poor nutritional status may impair immune responses and gut barrier function, potentially exacerbating mucosal inflammation [12,13]. In addition, clinically, fecal biomarkers also tie into nutritional consequences: chronic intestinal blood loss can contribute to iron-deficiency anemia, and severe inflammation can diminish appetite and nutrient absorption, worsening the patient’s nutritional status. Conversely, patients with poor nutrition (low protein stores or sarcopenia) may have less robust mucosal healing, thereby sustaining inflammation and bleeding [14]. Recent evidence indicates that IBD patients with sarcopenia or hypoalbuminemia tend to have higher inflammatory markers and more aggressive disease courses [15,16].

Although malnutrition is common in IBD and strongly associated with poorer outcomes, traditional disease severity scores often overlook nutritional status [14]. Given the interrelationships above, we hypothesize that IBD patients with poor nutritional status tend to exhibit higher FC levels and more frequent fecal occult blood, which correspond to increased disease activity. To explore this, the aim of our study is to investigate the relationship between disease activity and fecal markers (calprotectin and occult blood) in IBD patients, and to evaluate the role of nutritional status as measured by prognostic nutritional index (PNI) in this relationship.

## 2. Materials and Methods

This retrospective study was conducted on adult patients diagnosed with IBD and followed at the IBD Outpatient Clinic of the Anka Hospital between December 2023 and August 2025. The study adhered to the ethical regulations and principles specified in the Declaration of Helsinki and received approval from the Ethical Committee of Gaziantep City Hospital Clinical Research Ethics Committee (Date: 17 September 2025, Decision No: 286/2025). The requirement for obtaining informed consent was exempted by the Ethics Committee due to the retrospective design of the study.

### 2.1. Study Population

**Figure 1:**
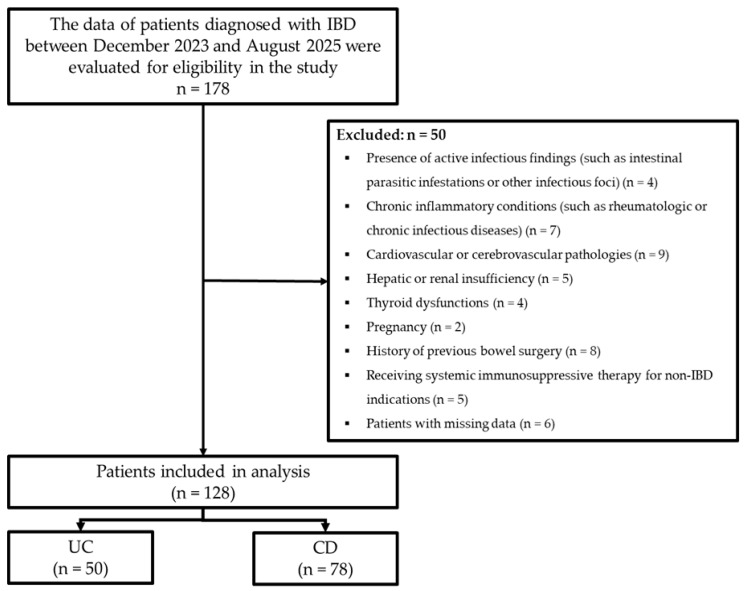
Flowchart of the study.

During the study period, a total of 178 patients diagnosed with IBD were retrospectively evaluated. Inclusion criteria comprised patients aged 18–95 years with a diagnosis of IBD, irrespective of gender. Exclusion criteria encompassed patients exhibiting active infectious findings (including intestinal parasitic infestations and/or other infective foci), chronic inflammatory conditions (such as rheumatologic or chronic infectious diseases), cardiovascular or cerebrovascular pathologies, hepatic or renal insufficiency, thyroid abnormalities, pregnancy, previous bowel surgery, and receipt of systemic immunosuppressive therapy for non-IBD indications (such as rheumatologic/oncologic conditions), and any individuals with missing data. After applying the exclusion criteria, the study comprised 128 IBD patients, including 50 with UC and 78 with CD (Figure 1).

### 2.2. Data Collection and Definitions

Demographic and clinical data were extracted from the hospital’s electronic information system and patient records. The dataset comprised patient age, gender, body mass index, smoking history, duration of illness, disease activity indices, pharmacotherapy regimens, laboratory values, fecal occult blood test results, and FC measurements.

To assess disease activity, a comprehensive history was taken at admission and all blood tests—including inflammatory markers—were performed. To ensure consistency in endoscopic assessment, all colonoscopic procedures were carried out by the same endoscopist with a FUJINON XL-4450 colonoscope (Fujifilm Medical Co., Tokyo, Japan). The severity of disease was determined by the Harvey–Bradshaw Index (HBI) in patients with CD and by the Mayo scoring system (Disease Activity Index, DAI) in those with UC [17,18]. Those with endoscopic activity index score of 5 or above were accepted to be an active disease.

Combination therapy was defined as the concurrent use of ≥2 systemic IBD agents at the index assessment—namely biologic plus immunomodulator (e.g., infliximab/adalimumab with azathioprine), biologic plus systemic corticosteroid (e.g., infliximab/adalimumab with prednisone), or immunomodulator plus systemic corticosteroid (e.g., azathioprine with prednisone).

### 2.3. Laboratory Measurements

Venous blood samples were obtained from all patients at their most recent hospital presentation after an 8 h fast, and analyzed using the Mindray MC6800 (Mindray, Shenzhen, China) and Architect Plus (Abbott Diagnostics, Abbott Park, IL, USA) systems. Leukocyte counts were obtained via impedance; C-reactive protein levels by immunoturbidimetry; and albumin by the bromocresol green method. The prognostic nutritional index (PNI) was calculated as: PNI = 10 × (albumin level in g/dL) + 0.005 × (total lymphocyte count) [19]. PNI values ≥ 50 were considered normal, while values < 50 were classified as low, indicating poor nutritional status [20,21,22].

FC was measured quantitatively using an enzyme-linked immunosorbent assay (ELISA) by IDS calprotectin extraction device (Immunodiagnostic Systems Ltd., Boldon, UK) [23]. Patients’ stool samples underwent fecal occult blood testing (FOBT) (within 20 h post-collection) using the HM-Jack autoanalyzer (Extel Hemo-Auto, Tokyo, Japan). The methodology relies on the agglutination of latex microparticles coated with anti-human hemoglobin antibodies upon interaction with hemoglobin, with resultant turbidity shifts quantitatively reflecting fecal blood levels. Test outcomes were classified as negative (0–12 ng/mL) or positive (>12 ng/mL).

### 2.4. Statistical Analysis

Statistical analyses were conducted in IBM SPSS Statistics for Windows (v. 20.0; IBM Corp., Armonk, NY, USA). Distributional normality was evaluated using the Kolmogorov–Smirnov test. Continuous variables conforming to a normal distribution are reported as mean ± SD; those deviating from normality are reported as median (IQR). Categorical data are presented as frequencies and percentages. Two-group comparisons of normally distributed continuous variables employed Student’s *t*-test, while one-way ANOVA was used for comparisons across three or more groups. For non-normally distributed continuous variables, the Mann–Whitney U test and Kruskal–Wallis H test were used for two-group and multi-group comparisons, respectively. Categorical comparisons utilized Pearson’s Chi-square or Fisher’s exact test based on expected cell counts. To determine potential predictors of disease activity scores, univariate linear regression analyses were performed. Parameters failing to meet normality assumptions underwent logarithmic transformation before inclusion in the multivariate linear regression model. Significance was accepted at *p* < 0.05 for all statistical analyses.

## 3. Results

A total of 128 IBD patients were analyzed, with 50 (39.1%) diagnosed with UC and 78 (60.9%) with CD. The mean age was 44.4 ± 14.1 years, and no significant difference was observed between UC and CD (42.7 ± 15.1 vs. 45.1 ± 13.7 years, *p* = 0.432). Females comprised 57% of the total population, with similar distributions in UC and CD (50.0% vs. 61.5%, *p* = 0.207). BMI, smoking status, and duration of illness were not significantly different between groups. Median disease activity score was higher in UC patients compared to CD patients (66.7% vs. 33.3%, *p* < 0.001). Regarding treatment, the use of ASA (52.0% vs. 20.5%, *p* < 0.001) was higher in UC patients, while biological agent use was higher in CD patients (14.1% vs. 0%, *p* = 0.030). No significant difference was found in the use of other therapies. Mean PNI level was lower in UC patients compared to CD patients (49.2 ± 4.2 vs. 51.5 ± 4.6, *p* = 0.048). Also, rate of poor nutrition status was higher in UC patients compared to CD patients (62.0% vs. 35.9%, *p* = 0.004). Positivity for FOBT was significantly greater in UC patients than in CD (76.7% vs. 43.6%, *p* = 0.002), and FC levels were markedly higher in UC patients compared to CD (278.0 vs. 133.5 µg/g, *p* < 0.001) (Table 1).

In patients with UC, disease activity scores showed significant positive correlations with duration of illness (r = 0.380, *p* < 0.001), leukocyte count (r = 0.326, *p* = 0.029), neutrophils count (r = 0.349, *p* < 0.001), CRP levels (r = 0.376, *p* < 0.001), and FC levels (r = 0.675, *p* < 0.001). In contrast, significant negative correlations were observed with albumin levels (r = −0.371, *p* < 0.001) and PNI values (r = −0.420, *p* < 0.001). Additionally, poor nutritional status and FOBT positivity were associated with higher disease activity scores (*p* < 0.05). Similar associations were observed in patients with CD patient (Table 2).

**Figure 2:**
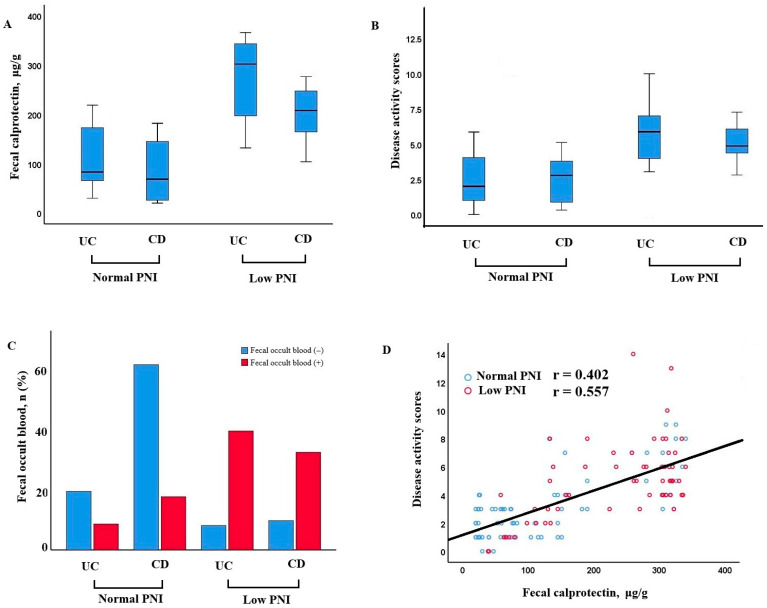
Relationship between nutritional status, fecal calprotectin, disease activity, and occult blood in UC and CD patients. (**A**) Fecal calprotectin levels in patients with UC and CD, stratified by normal and low PNI. Patients with low PNI exhibited higher calprotectin levels, particularly in the CD group. (**B**) Disease activity scores in UC and CD patients according to PNI status. Higher scores were observed in the low PNI groups. (**C**) Distribution of fecal occult blood positivity in UC and CD patients with normal and low PNI. Fecal occult blood was more frequently positive in patients with low PNI, especially in CD. (**D**) Correlation between fecal calprotectin and disease activity scores. A positive association was identified, more prominent among patients with low PNI.

Patients with low PNI demonstrated significantly higher fecal calprotectin levels and disease activity scores in both UC and CD groups. Additionally, fecal occult blood positivity was more frequent in patients with low PNI. A positive correlation was observed between fecal calprotectin and disease activity, especially in those with poor nutritional status (r = 0.402, *p* < 0.001 for normal nutrition; r = 0.557, *p* < 0.001 for poor nutrition) (Figure 2).

Variables significantly associated with disease activity scores in univariable analysis are presented in Table 3. Multivariable regression analysis was performed to determine independent predictors of disease activity, and results are shown as Model I and Model II. In Model I, all potential risk factors except FOBT positivity and FC were included. Model II incorporated the FOBT positivity and FC in addition to those in Model I. All models were adjusted for age, sex, BMI, smoking status, and medication effects. For UC, Model I included log(Duration of illness), log(CRP), and PNI. All variables emerged as independent predictors: longer duration of illness (β = 0.43, 95% CI: 0.20–0.68, *p* = 0.001), higher CRP levels (β = 0.20, 95% CI: 0.04–0.36, *p* = 0.014), and lower PNI values (β = −0.03, 95% CI: −0.04 to −0.01, *p* = 0.008). Model II included log(Duration of illness), log(CRP), fecal occult blood, and log(Fecal calprotectin), revealing that longer illness duration (β = 0.48, 95% CI: 0.18–0.78, *p* = 0.003), higher CRP (β = 0.13, 95% CI: 0.04–0.22, *p* = 0.009), FOBT positivity (β = 0.34, 95% CI: 0.13–0.55, *p* = 0.002), and elevated FC (β = 0.91, 95% CI: 0.68–1.14, *p* < 0.001) remained independent predictors (Table 3). Similar analyses for CD in Model I identified log(Duration of illness) (β = 0.30, 95% CI: 0.14–0.47, *p* < 0.001), log(CRP) (β = 0.18, 95% CI: 0.03–0.32, *p* = 0.018), and lower PNI (β = −0.02, 95% CI: −0.03 to −0.01, *p* = 0.013) as significant independent predictors. Model II confirmed log(Duration of illness) (β = 0.27, 95% CI: 0.13–0.42, *p* < 0.001), log(CRP) (β = 0.12, 95% CI: 0.02–0.25, *p* = 0.042), FOBT positivity (β = 0.19, 95% CI: 0.07–0.32, *p* = 0.002), and elevated FC (β = 0.33, 95% CI: 0.20–0.46, *p* < 0.001) as independent predictors of disease activity (Table 3).

## 4. Discussion

In this study of patients with IBD, several key biomarkers were found to correlate with disease activity. Fecal calprotectin (FC) levels were significantly elevated in patients with active IBD compared to those in remission, reflecting a higher inflammatory burden in the gut. Similarly, a greater proportion of patients in active disease had a positive FOBT, indicating occult gastrointestinal bleeding associated with mucosal ulceration. Markers of systemic inflammation were also higher during active disease—patients in flare showed elevated CRP levels and often had increased leukocytes. In contrast, nutritional status indicators were poorer in active IBD: serum albumin was significantly lower and the PNI was depressed, consistent with malnutrition and systemic inflammation. Notably, PNI independently predicted disease activity in the initial model but lost significance upon inclusion of FC and FOBT, suggesting that nutritional status may indirectly influence disease activity via these fecal biomarkers.

Numerous studies confirm that FC correlates strongly with IBD activity [24,25,26,27]. A study involving patients with UC and CD reported that fecal calprotectin levels exhibited sensitivity greater than 90% and specificity greater than 80% in differentiating active disease from remission [24]. Consistent with these findings, a strong correlation was observed between IBD activity and FC. Nevertheless, compared with Crohn’s disease, ulcerative colitis involved a higher proportion of patients in an active state [28], who also demonstrated increased FC levels. There may be several potential mechanisms underlying this condition. In active UC, neutrophils infiltrate the colonic mucosa and release calprotectin into the lumen, so stool FC levels rise in proportion to inflammatory burden [29]. However, CD can involve patchy, transmural inflammation and segments of small intestine where fecal markers may be less uniformly elevated [30]. Another important mechanism may be the higher proportion of FOBT-positive patients in UC than in those with UC [31]. Rectal bleeding (hematochezia) is a hallmark symptom of active UC, so it is unsurprising that FOBT frequently turn positive during UC flares. Comparative studies highlight this difference: in UC, fecal immunochemical test (FIT) and FC perform equally well in detecting mucosal disease, but in CD, FIT is markedly less sensitive for small-bowel lesions compared to FC [32]. This is because CD inflammation that is confined to the ileum or jejunum may not lead to substantial bleeding into feces, or blood is degraded during transit. The fecal immunochemical test “misses” purely inflammatory lesions if they do not bleed, whereas FC will still be elevated from neutrophil influx [32]. These findings suggest that both FOBT positivity and FC may be important predictors in UC patients, whereas in CD patients, FC may serve as a more important marker than FOBT.

An additional noteworthy factor may involve the nutritional status of IBD patients. It is estimated that 20–85% of IBD patients suffer from malnutrition at some point, with the prevalence on the higher end (up to ~75%) during active disease flares [33]. Although clinical remission and the absence of symptoms do not necessitate dietary restrictions, studies have reported that macronutrient and fiber intake in IBD patients differs from that of healthy individuals, with no significant differences observed between CD and UC patients in terms of total caloric, protein, lipid, carbohydrate, or fiber intake [34]. Despite this, malnutrition tends to be more pronounced in patients with CD. Because CD can affect extensive portions of the GI tract, it more commonly leads to protein–energy malnutrition and specific nutrient deficiencies than UC [35,36]. In UC, malabsorption is less pronounced, but chronic blood loss and inflammation can still lead to deficiencies (e.g., iron, vitamin D) and protein-losing enteropathy [33]. The inflammatory state itself exacerbates malnutrition by increasing catabolism and muscle breakdown, as well as by inducing anorexia and altering gut hormone signals that regulate appetite and metabolism [14]. This may explain the lower PNI values and the stronger correlation with FC observed in UC patients, who had a higher rate of active disease.

Locally, inflammation also disrupts the epithelial barrier and absorptive function of the gut. The inflamed mucosa has blunted villi and increased permeability (“leaky gut”), reducing the surface area and integrity needed for nutrient absorption [36,37]. Tight junctions between cells are weakened, allowing microbial products and proteins to leak across, which in turn sustains immune activation and can worsen systemic inflammation [33]. In the intestinal mucosa, neutrophils flood into the tissue and lumen, drawn by chemokines. These activated neutrophils release calprotectin (among other proteins), which explains why fecal calprotectin rises in proportion to neutrophilic inflammation. Essentially, FC is a direct readout of neutrophil activity at the mucosal surface [38]. Neutrophils also release myeloperoxidase, elastase, and other enzymes that contribute to mucosal injury, potentially leading to erosions and ulcers [39,40]. When ulcers breach the submucosa, capillaries are exposed and bleed—resulting in blood entering the stool. Accordingly, FC—a proximal index of mucosal neutrophilic inflammation—exhibits a more immediate and specific correspondence with disease activity, whereas PNI, which is depressed via inflammation-induced malabsorption and negative acute-phase responses, represents a downstream systemic consequence. Hence, PNI may correlate with disease activity when considered alone (Model 1 regression analysis), but typically loses independent significance after adjustment for FC (Model 2 regression analysis), consistent with FC capturing variance more causally proximate to the inflammatory disease process.

### 4.1. Limitations

This study has several limitations. First, the retrospective nature of the study may have introduced selection bias and restricted the ability to draw causal inferences. Additionally, this was a single-center study. Between-hospital variability in stool-biomarker analytics (e.g., brand- and lot-specific FIT/FOBT and fecal calprotectin assays) and differences in local therapeutic algorithms may influence absolute values and operating characteristics [41,42]. Moreover, regional or geographic variability may contribute to differences in the sociodemographic profiles of populations, which play an important role in shaping IBD phenotypes and malnutrition risk [43,44,45]. These factors may limit the generalizability of the study findings to community-based or multiethnic cohorts. Second, disease activity was assessed by symptom-based indices (Partial Mayo for UC and HBI for CD) rather than uniformly paired endoscopic or histologic measures, which may lead to misclassification. Third, phenotypic characterization based on the Montreal classification (e.g., UC extent, CD location and behavior) was not systematically included due to incomplete documentation in the retrospective records. As such, the potential influence of disease phenotype on biomarker levels and nutritional status could not be fully analyzed. Research indicates that fecal calprotectin’s accuracy in CD can be influenced by disease location: colonic CD behaves similarly to UC with high FC levels, whereas isolated small-bowel CD might produce somewhat lower FC despite active inflammation [46]. Finally, it lacked longitudinal follow-up, precluding assessment of changes in fecal marker levels or nutritional status over time.

### 4.2. Future Directions

Future prospective, multi-center studies with larger, phenotype-stratified cohorts that directly address these limitations are warranted to validate our findings and to determine whether nutrition-focused interventions, coupled with biomarker-guided treat-to-target care, can disrupt the inflammation–malnutrition cycle and improve outcomes. Emerging evidence suggests that integrating stool biomarkers with nutritional indices could enable a more personalized approach to IBD management moving forward. Biomarker-guided nutritional interventions may represent a promising strategy. An elevated FC or positive FOBT in a stable patient could prompt earlier dietary support (such as exclusive enteral nutrition or targeted supplementation) to preempt an impending flare [47,48]. Likewise, trends such as a declining PNI or persistent hypoalbuminemia might signal deteriorating nutritional status and justify intensifying nutritional interventions in anticipation of clinical deterioration [14]. In parallel, these biomarkers offer value for clinical risk stratification. Patients with persistently high FC or recurrent FOBT positivity are at heightened risk of relapse and complications, and combining such stool markers with indices of nutritional status can identify high-risk individuals who warrant closer monitoring and aggressive, multidisciplinary management. Notably, malnourished IBD patients (e.g., those with sarcopenia or low PNI) tend to experience more active disease and worse outcomes, underscoring the importance of early nutritional optimization as part of the care plan [14]. Furthermore, current guidelines issued by organizations such as the European Crohn’s and Colitis Organization (ECCO) and ESPEN emphasize the importance of routine nutritional risk screening and support in the management of IBD [49]. In addition, treat-to-target strategies—such as STRIDE—incorporate objective biomarkers like FC to guide proactive treatment adjustments before irreversible tissue damage occurs [50,51]. Therefore, future research and clinical guidelines may provide direction on how stool biomarkers and nutritional indices can be used to guide the timing and intensity of both medical and nutritional interventions. This approach may ultimately help disrupt the inflammation–malnutrition cycle, prevent impending flares, and improve long-term clinical outcomes.

## 5. Conclusions

In these IBD patients, disease activity scores were associated with higher fecal biomarker burden and more pronounced nutritional impairment. In both the UC and CD groups, patients with malnutrition exhibited higher fecal biomarker burden. In both groups, fecal biomarkers remained an independent indicator of disease activity in the regression model, whereas PNI lost its significance. These findings suggest that malnutrition is a consequence of severe mucosal inflammation rather than an independent trigger for disease flare. Integrating fecal biomarkers into routine IBD monitoring, while utilizing PNI as a supplementary risk indicator, may offer a simple and noninvasive strategy for stratifying patients by flare risk. In practice, regular FC and FOBT assessments can help identify those at higher risk of an impending flare, prompting clinicians to initiate timely therapeutic escalation and provide targeted nutritional support. Adopting this pragmatic, biomarker-first approach with PNI as an adjunct offers a path to earlier escalation and tailored nutrition care, mitigating flare risk and enhancing long-term clinical trajectories.

## Figures and Tables

**Table 1:** Distribution of demographic characteristics and clinical findings.

Variables	All Population	Ulcerative Colitis	Crohn’s Disease	*p*
	*n* = 128	*n* = 50	*n* = 78	
Age, years	44.4 ± 14.1	42.7 ± 15.1	45.1 ± 13.7	0.432
Female gender, *n* (%)	71 (55.5)	25 (50.0)	46 (59.0)	0.364
BMI, kg/m^2^	26.0 ± 4.9	26.1 ± 4.7	25.9 ± 5.0	0.878
Smoking, *n* (%)	30 (23.4)	14 (28.0)	16 (20.5)	0.394
Duration of illness, years	6.0 (4.0–10.0)	8.5 (4.2–10.0)	6.0 (4.0–10.0)	0.401
Disease activity scores	4.0 (2.0–6.0)	5.0 (3.2–7.0)	3.0 (2.0–5.0)	0.002 *
Inactive, *n* (%)	75 (58.6)	13 (26.0)	67 (66.7)	<0.001 *
Activity, *n* (%)	53 (41.4)	27 (74.0)	26 (33.3)	
Drugs, *n* (%)				
ASA	40 (31.3)	24 (48.0)	16 (20.5)	0.002 *
Azathioprine	27 (21.1)	10 (20.0)	17 (21.8)	0.999
IFX/ADA	11 (8.6)	–	11 (14.1)	0.007 *
Combination therapy	52 (40.6)	18 (36.0)	34 (43.6)	0.462
Laboratory findings				
Hemoglobin, g/dL	13.1 ± 1.2	13.2 ± 1.3	13.1 ± 1.1	0.679
Leukocytes, ×10^3^ µL	8.2 ± 1.9	8.3 ± 1.4	8.1 ± 2.0	0.518
Platelets, ×10^3^ µL	287.8 ± 59.6	301.2 ± 65.5	282.7 ± 56.8	0.148
Neutrophils, ×10^3^ µL	5.5 ± 1.5	5.7 ± 1.3	5.3 ± 1.6	0.043 *
Lymphocytes, ×10^3^ µL	1.8 (1.3–2.2)	1.6 (1.3–2.0)	1.9 (1.5–2.2)	0.023 *
Glucose, md/dL	93.8 ± 15.4	96.1 ± 14.8	92.9 ± 15.6	0.337
Creatinine, mg/dL	0.8 ± 0.2	0.8 ± 0.2	0.8 ± 0.2	0.973
AST, IU/L	18.0 (16.0–22.0)	18.0 (16.0–21.8)	18.0 (16.0–22.0)	0.553
ALT, IU/L	14.0 (11.0–21.2)	14.0 (11.2–19.8)	14.0 (11.2–22.0)	0.812
CRP, mg/dL	7.0 (2.7–11.0)	10.0 (3.5–15.0)	6.7 (3.0–10.0)	0.072
Albumin, g/L	4.1 ± 0.3	4.0 ± 0.4	4.2 ± 0.3	0.036 *
PNI	50.1 ± 4.8	49.2 ± 4.2	51.5 ± 4.6	0.048 *
Poor nutrition, *n* (%)	59 (62.0)	31 (62.0)	28 (35.9)	0.006 *
Fecal occult blood, *n* (%)	57 (52.8)	23 (76.7)	34 (43.6)	0.002 *
Fecal calprotectin, μg/g	156.0 (64.0–310.0)	278.0 (200.0–321.5)	133.5 (50.8–280.8)	<0.001 *

Data are mean ± standard deviation or median (IQR) or number (%). Combination therapy was defined as the concurrent use of ≥2 systemic IBD agents at the index assessment—namely biologic plus immunomodulator (e.g., infliximab/adalimumab with azathioprine), biologic plus systemic corticosteroid (e.g., infliximab/adalimumab with prednisone), or immunomodulator plus systemic corticosteroid (e.g., azathioprine with prednisone). PNI values ≥ 50 were considered normal, while values < 5 0 were classified as low, indicating poor nutritional status. * *p* < 0.05 indicates statistical significance. Abbreviations: ADA, adalimumab; AST, aspartate aminotransferase; ALT, alanine aminotransferase; BMI, body mass index; CRP, C-reactive protein; IFX, infliximab; PNI, prognostic nutritional index.

**Table 2:** Findings associated with disease activity score.

Variables	Ulcerative Colitis		Crohn’s Disease	
	Median (IQR) or Correlation Coefficient (r)	*p*	Median (IQR) or Correlation Coefficient (r)	*p*
Age	r = −0.008	0.966	r = −0.099	0.387
Sex				
Female	5 (2–7)	0.267	3 (2–5)	0.318
Male	6 (4–8)		4 (3–5)	
BMI	r = 0.064	0.738	r = 0.090	0.433
Smoking				
No	5 (2–8)	0.836	3 (2–5)	0.470
Yes	6 (4–7)		3 (2–5)	
Duration of illness	r = 0.380	<0.001 *	r = 0.322	<0.001 *
Drugs				
ASA	5 (3–7)	0.223	3 (2–4)	0.251
Azathioprine	4 (3–6)		3 (1–5)	
IFX/ADA	-		3 (2–4)	
Combination therapy	4 (2–5)		4 (3–5)	
Laboratory findings				
Hemoglobin	r = −0.113	0.554	r = −0.006	0.955
Leukocytes	r = 0.356	<0.001 *	r = 0.368	<0.001 *
Platelets	r = 0.236	0.402	r = 0.113	0.326
Neutrophils	r = 0.349	<0.001 *	r = 0.357	<0.001 *
Lymphocytes	r = −0.375	<0.001 *	r = −0.366	<0.001 *
Glucose	r = 0.066	0.730	r = 0.036	0.752
Creatinine	r = −0.252	0.293	r = 0.151	0.387
AST	r = 0.223	0.382	r = −0.097	0.420
ALT	r = 0.216	0.251	r = −0.162	0.156
CRP	r = 0.376	<0.001 *	r = 0.367	<0.001 *
Albumin	r = −0.371	<0.001 *	r = −0.332	0.003 *
PNI	r = −0.420	<0.001 *	r = −0.441	<0.001 *
Nutrition status				
Normal	3 (2–5)	0.019 *	3 (1–4)	<0.001 *
Poor	6 (4–7)		5 (4–6)	
Fecal occult blood				
No	2 (1–2)	<0.001 *	3 (1–4)	0.011 *
Yes	6 (4–8)		5 (3–6)	
Fecal calprotectin	r = 0.675	<0.001 *	r = 0.630	<0.001 *

Combination therapy was defined as the concurrent use of ≥2 systemic IBD agents at the index assessment—namely biologic plus immunomodulator (e.g., infliximab/adalimumab with azathioprine), biologic plus systemic corticosteroid (e.g., infliximab/adalimumab with prednisone), or immunomodulator plus systemic corticosteroid (e.g., azathioprine with prednisone). PNI values ≥ 50 were considered normal, while values < 50 were classified as low, indicating poor nutritional status. * *p* < 0.05 indicates statistical significance. Abbreviations: ADA, adalimumab; AST, aspartate aminotransferase; ALT, alanine aminotransferase; BMI, body mass index; CRP, C-reactive protein; IFX, infliximab; PNI, prognostic nutritional index.

**Table 3:** Independent predictors for disease activity score in IBD patients.

Variables	Crude Regression			Model I Regression			Model II Regression		
	β ± SE	95% CI	*p*	β ± SE	95% CI	*p*	β ± SE	95% CI	*p*
Ulcerative colitis									
log(Duration of illness)	0.44 ± 0.14	0.15–0.73	<0.001 *	0.43 ± 0.11	0.20–0.68	0.001 *	0.48 ± 0.15	0.18–0.78	0.003 *
Leukocytes	0.08 ± 0.04	0.01–0.16	0.029 *	0.03 ± 0.03	(−0.04)–0.10	0.371	0.03 ± 0.02	(−0.10)–0.06	0.158
log(CRP)	0.23 ± 0.08	0.06–0.39	<0.001 *	0.20 ± 0.07	0.04–0.36	0.014 *	0.13 ± 0.05	0.04–0.22	0.009 *
PNI	−0.03 ± 0.01	(−0.05)–(−0.01)	<0.001 *	−0.03 ± 0.01	(−0.04)–(−0.01)	0.008 *	0.01 ± 0.01	(−0.01)–0.02	0.150
Fecal occult blood	0.49 ± 0.08	0.31–0.66	<0.001 *	not included			0.34 ± 0.10	0.13–0.55	0.002 *
log(Fecal calprotectin)	1.06 ± 0.14	0.78–1.34	<0.001 *	not included			0.91 ± 0.11	0.68–1.14	<0.001 *
				Adjusted R^2^ = 0.38			Adjusted R^2^ = 0.52		
Crohn’s disease									
log(Duration of illness)	0.23 ± 0.09	0.04–0.41	<0.001 *	0.30 ± 0.08	0.14–0.47	<0.001 *	0.27 ± 0.07	0.13–0.42	<0.001 *
Leukocytes	0.05 ± 0.02	0.02–0.18	0.023 *	0.08 ± 0.07	(−0.05)–0.23	0.220	0.02 ± 0.01	(−0.01)–0.05	0.110
log(CRP)	0.28 ± 0.07	0.14–0.43	<0.001 *	0.18 ± 0.07	0.03–0.32	0.018 *	0.12 ± 0.06	0.02–0.25	0.042 *
PNI	−0.02 ± 0.01	(−0.03)–(−0.01)	<0.001 *	−0.02 ± 0.01	(−0.03)–(−0.01)	0.013 *	−0.01 ± 0.01	(−0.03)–0.01	0.107
Fecal occult blood	0.29 ± 0.06	0.17–0.40	0.011 *	not included			0.19 ± 0.06	0.07–0.32	0.018 *
log(Fecal calprotectin)	0.40 ± 0.07	0.27–0.52	<0.001 *	not included			0.43 ± 0.06	0.30–0.56	<0.001 *
				Adjusted R^2^ = 0.32			Adjusted R^2^ = 0.45		

Logarithmic transformation was applied to numerical variables that did not exhibit normal distribution, such as disease activity score. * *p* < 0.05 indicates statistical significance. Abbreviations: β, unstandardized regression coefficients; CI, Confidence intervals; CRP, C-reactive protein; PNI, prognostic nutritional index; SE: standard error.

## Data Availability

The data that support the findings of this study are available on request from the corresponding author.
